# Blood metabolomic profiling predicts postoperative gastrointestinal function of colorectal surgical patients under the guidance of goal-directed fluid therapy

**DOI:** 10.18632/aging.202711

**Published:** 2021-03-10

**Authors:** Tao Xie, Zhengyu Jiang, Cen Wen, Du Shen, Jinjun Bian, Shanshan Liu, Xiaoming Deng, Yanping Zha

**Affiliations:** 1 Faculty of Anesthesiology, Changhai Hospital, Naval Medical University/ Second Military Medical University, PLA, Shanghai 200433, China; 2 Department of Anesthesiology, Naval Medical Center, Naval Medical University/ Second Military Medical University, PLA, Shanghai 200052, China; 3 Department of Anesthesiology, Sichuan Academy of Medical Sciences and Sichuan Provincial People's Hospital, University of Electronic Science and Technology of China, Chengdu 610072, China; 4 Department of Anesthesiology, The Affiliated Hospital of Medical School, Ningbo University, Ningbo 315020, China; 5 Department of Anesthesiology, Chenggong Hospital, Xiamen University, Xiamen 361001, China

**Keywords:** colorectal surgery, metabolomics, goal-directed fluid therapy, gastrointestinal function

## Abstract

Postoperative gastrointestinal function influences postoperative recovery and length of hospital stay for patients undergoing colorectal surgery. Goal-directed fluid therapy (GDFT) restricts fluid administration to an amount required to prevent dehydration. Although the fluid management of GDFT could decrease the incidence of postoperative complications in patients who undergo high-risk surgery, certain patients may not respond to GDFT. Thus, to achieve optimal treatment, identification of patients suitable for GDFT is necessary. Metabolomic profiling of 48 patients undergoing surgery for colorectal cancer was performed. Patients were divided into delayed- and enhanced-recovered groups based on gastrointestinal function within 72 hours, and the results of omics analysis showed differential serum metabolites between the two groups of patients in the post anesthesia care unit 24 hours after surgery. A support vector machine model was applied to evaluate the curative effects of GDFT in different patients. Four metabolites, oleamide, ubiquinone-1, acetylcholine, and oleic acid, were found to be highly associated with postoperative gastrointestinal function and could be used as potential biomarkers. Moreover, four pathways were found to be highly related to postoperative gastrointestinal recovery. Among them, the vitamin B6 metabolism pathway may be a common pathway for improving postoperative recovery in various diseases. Our findings proposed a novel method to predict postoperative recovery of gastrointestinal function based on metabolomic profiling and suggested the potential mechanisms contributing to gastrointestinal function after surgical resection of colorectal cancer under the fluid management of GDFT.

## INTRODUCTION

Postoperative gastrointestinal (GI) recovery significantly influences perioperative complications and prognosis in patients undergoing colorectal surgery, particularly for elderly patients who are at risk of increased hospital stay and organ dysfunction owing to delayed GI recovery [1]. The amount of intravenous (i.v.) fluid administration is related to perioperative morbidity, and excess or insufficient fluid delivery can increase postoperative complications [2, 3]. Traditional i.v. therapy is usually determined by the perceived magnitude of surgical trauma without the supporting physiological principles [4]. Therefore, i.v. therapy might be harmful if patients do not respond to fluid delivery or do not exhibit enhanced blood flow upon fluid administration [5]. Goal-directed fluid therapy (GDFT) based on the Enhanced Recovery After Surgery (ERAS) program restricts fluid administration to an amount required to prevent dehydration, both during and after surgery [6, 7]. Studies have shown that GDFT decreases the incidence of postoperative complications in patients who undergo high-risk surgery [8, 9].

By adjusting arterial pressure and blood flow parameters, including cardiac output, cardiac index, stroke volume index, stroke volume (SV) variation (SVV), and oxygen delivery, GDFT can modify perioperative fluid administration to achieve suitable end-organ perfusion [7]. SVV is used to reflect variations in left ventricular output caused by changes in intrathoracic pressure induced by mechanical ventilation, representing the percentage of changes between the maximum and minimum values of SVs over a period of time divided by their mean values. SVV has been shown to be an accurate predictor of fluid responsiveness [10] and could be used as a resuscitation endpoint to evaluate postoperative recovery.

The ERAS program has facilitated progress in promoting the recovery of intestinal function and has been proposed as a standard strategy for enhancement of postoperative recovery in northern Europe and other countries [11, 12]. However, there are few studies to predict and evaluate the postoperative GI function of patients with colorectal surgery, and usually only clinical outcomes are used to evaluate whether the postoperative GI function of patients is well restored. Therefore, it is necessary to identify the measures that could predict the possible long-term postoperative recovery condition of GI function in patients at the immediate time point or early after surgery, and to implement early intervention strategies for patients with poor postoperative GI function recovery.

Metabolomics is a novel approach with many applications in biological research. With the development of advanced analytical techniques and bioinformatics, metabolomics has been extensively applied as a holistic diagnostic tool in clinical and biomedical studies. In contrast to proteomics, genomics, and other methods that reflect indirect changes in the system, metabolomics reveals the direct response of the physiological environment [13]. In the case of postoperative recovery of intestinal function, which develops asymptotically, metabolite changes can occur much earlier in the postoperative period. Metabolomics can reflect the influence of surgical trauma and anesthesia on the general condition of patients during the early postoperative period. However, whether metabolic factors affect the recovery of intestinal function or its predictive effect on postoperative recovery after colorectal surgery has not been extensively studied.

Recently, researchers have demonstrated that some patients exhibit poor postoperative recovery condition under the intra-operative fluid management of GDFT [14]. Therefore, in this study, we aimed to further identify the influence of GDFT on the recovery of postoperative GI function in patients who underwent colorectal resection, and to explore potential biomarkers that could predict the possible long-term recovery condition of GI function in patients by metabolomics analysis of plasma samples early after surgery.

## RESULTS

### Basic characteristics of patients in the delayed and enhanced postoperative recovery groups

The demographic data for all patients were shown in Table 1. Among 48 patients with colorectal cancer, 24 showed enhanced postoperative recovery after surgery. There were no significant differences between the enhanced group and the delayed group in age, sex, BMI, ASA grade, operation duration, preoperative SVV, postoperative SVV, preoperative mean blood pressure (MBP), postoperative MBP, intra-operative fluid administration, perioperative 24-hour fluid administration and other aspects. In the enhanced group, the time of flatus, defecation, and feeding was advanced, the incidence of anemia was reduced, and the incidence of postoperative nausea and vomiting was decreased.

**Table 1:** Characteristics of the study population.

	**Enhanced (n = 24)**	**Delayed (n = 24)**	***P* value**
**Baseline**			
Age (years)	56.04 ± 5.58	53.88 ± 8.41	0.2
Female sex	9	9	1
BMI	22.67 ± 2.06	22.5 ± 2.22	0.772
ASA (I/II/III)	6/16/2	3/20/1	
**Comorbidities**			
Hypertension	3	0	0.234
**Type of surgery**			
Anterior resection of rectum	9	6	0.534
Left hemicolectomy	4	6	0.724
Right hemicolectomy	3	4	> 0.999
Transverse colectomy	4	3	> 0.999
Other	4	5	> 0.999
**Intra-operative data**			
Operation duration (min)	142.5 ± 54.39	133.9 ± 45.34	0.562
SVV (before surgery)	7.96 ± 1.88	8.21 ± 1.96	0.651
SVV (after surgery)	6.29 ± 1.65	6.42 ± 1.74	0.803
MBP (before surgery)	77.67 ± 7.11	75.63 ± 8.81	0.381
MBP (after surgery)	83.46 ± 7.84	86.63 ± 10.64	0.247
Intra-operative fluids	1505 ± 468.4	1520 ± 497.7	0.915
24 h perioperative fluids	3403 ± 622	3415 ± 165.2	0.956
**Outcomes**			
Flatus time	1.83 ± 0.38	3.63 ± 1.35	< 0.001
Defecation	3.46 ± 1.50	5.13 ± 1.39	< 0.001
Feeding time	3.17 ± 1.31	4.71 ± 2.65	0.014
Hospital stay (days)	9.58 ± 1.95	10 ± 3.02	0.572
**Complications**			
Ileus	1	2	> 0.999
Fistula	0	1	> 0.999
Incision infection	2	2	> 0.999
Anemia	0	5	0.049
Postoperative nausea	4	7	0.049
Postoperative vomiting	1	4	0.0028

Abbreviations: BMI, body mass index; ASA, American Society of Anesthesiologists classification; CAD, coronary artery disease; SVV, stroke volume variation; MBP, mean blood pressure.

### Overall metabolomics analysis of blood samples

**Figure 1:**
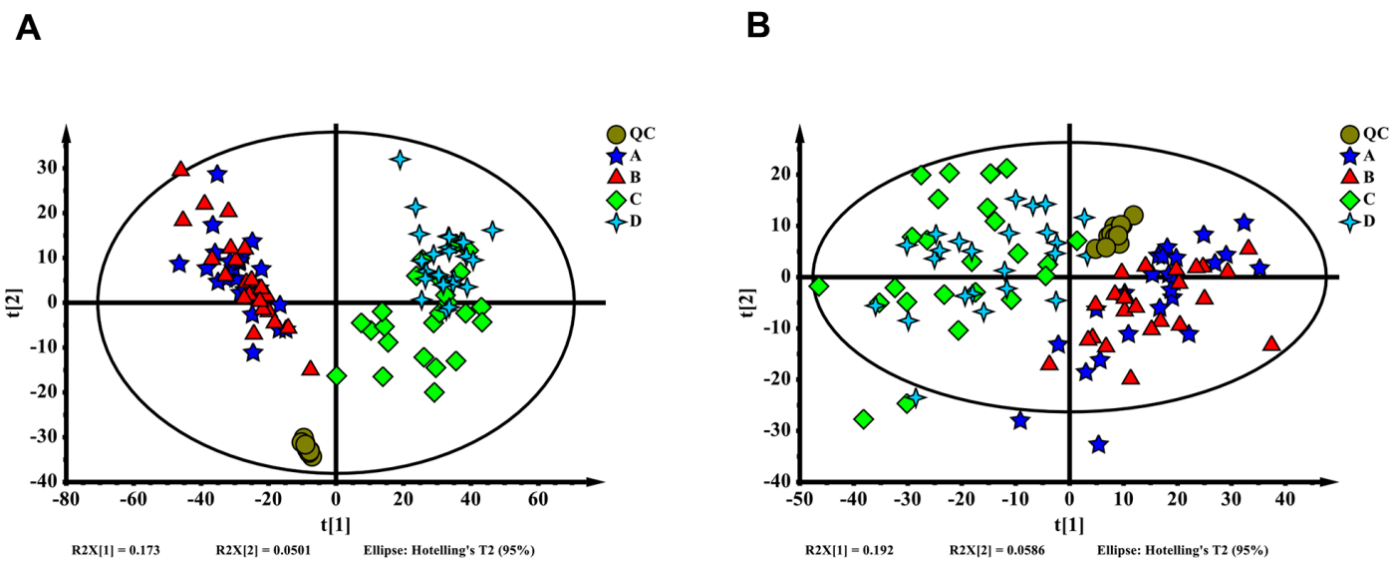
(**A**) Orthogonal partial least squares discriminant analysis (OPLS-DA) score plot of all samples (ESI+). (**B**) OPLS-DA score plot of all samples (ESI-). The QC, A, B, C and D represent the sample group in each mode, delayed PACU samples were A, enhanced PACU samples were B, delayed 24 hours samples were C, and enhanced 24 hours samples were D.

The metabolomics of 96 blood samples from 48 patients who underwent surgery for colorectal cancer in the enhanced and delayed groups were characterized and compared. Blood samples were collected when patients were in the post anesthesia care unit (PACU) and at 24 hours after surgery for all 48 patients. Delayed PACU samples were separated as group A, enhanced PACU samples were separated as group B, delayed 24 hours samples were separated as group C, and enhanced 24 hours samples were separated as group D. Representative ESI+ and ESI- ion chromatograms for each group are presented in Supplementary Figure 1. In total, 4,652 molecular features in ESI+ ion mode and 1,898 molecular features in ESI- ion mode were obtained and subjected to MVA. Principal component analysis extracted 16 principal components for ESI+ ion mode with a Q^2^ of 0.255 and R^2^X of 0.553. For ESI- ion mode (Figure 1A), there were 15 principal components, with a Q^2^ of 0.284 and R^2^X of 0.584 (Figure 1B). From the overall point of view, enhanced and delayed group distributions were overlapped in both ESI+ ion mode and ESI- ion mode, and a significant difference existed between PACU and 24 hours after surgery groups.

**Figure 2:**
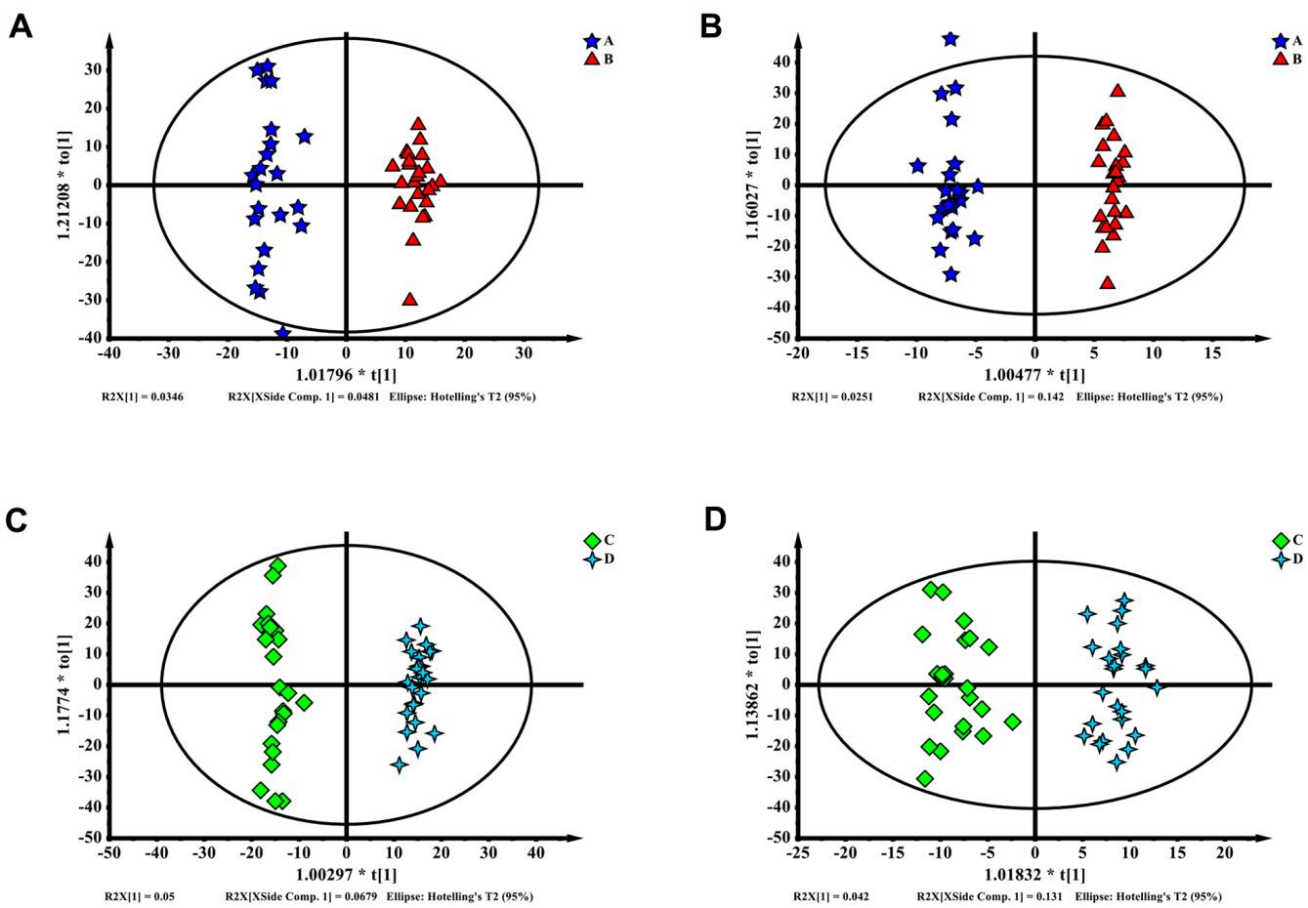
(**A**–**D**) Partial least squares discriminant analysis (PLS-DA) of blood metabolomics data from delayed (**A**, **C**) and enhanced (**B**, **D**) postoperative recovery groups in PACU (**A**, **B**) or postoperative 24 hours (**C**, **D**). Blood metabolites were used to distinguish between patients with different postoperative recovery outcomes.

The partial least squares discriminant analysis (PLS-DA) results of enhanced and delayed postoperative recovery samples in the PACU revealed differences in their distributions, with a Q^2^ of 0.343 and R^2^Y of 0.97 in ESI+ ion mode (Figure 2A) and a Q^2^ of 0.433 and R^2^Y of 0.95 in ESI- ion mode (Figure 2B). Although the R^2^Y reflected a not over lifting model, the Q^2^ had low prediction rate, suggesting that this model may be unreliable. Features with variable importance in projection (VIP) scores greater than 1.0 in multivariate statistical analysis and *p* values of less than 0.05 in univariate analysis were considered as the most significant metabolites. Twenty metabolites for ESI+ ion mode and eight metabolites for ESI- ion mode were selected as the most significant differentiating metabolites (Supplementary Table 1).

The PLS-DA results for blood samples collected 24 hours after surgery in the enhanced and delayed postoperative recovery patients revealed differences in their distributions, with a Q^2^ of 0.691 and R^2^Y of 0.985 for ESI+ ion mode (Figure 2C) and a Q^2^ of 0.453 and R^2^Y of 0.811 for ESI- mode (Figure 2D). This model also appeared to have a low prediction rate and to be not over lifting. Features with VIP scores greater than 1.0 in multivariate statistical analysis and *p* values less than 0.05 in univariate analysis were considered as the most significant metabolites. In total, 27 metabolites for ESI+ ion mode and seven metabolites for ESI- ion mode were selected as the most significant differentiating metabolites (Supplementary Table 2).

In a comparison of differential metabolites in groups A–B and C–D, we found similar variation tendencies. The differential metabolites are shown in Supplementary Tables 1, 2. Most of the metabolites showing similar trends were not identified as significant differential metabolites. However, phosphohydroxypyruvic acid rates were high in groups A–B and C–D in ESI+ ion mode.

**Figure 3:**
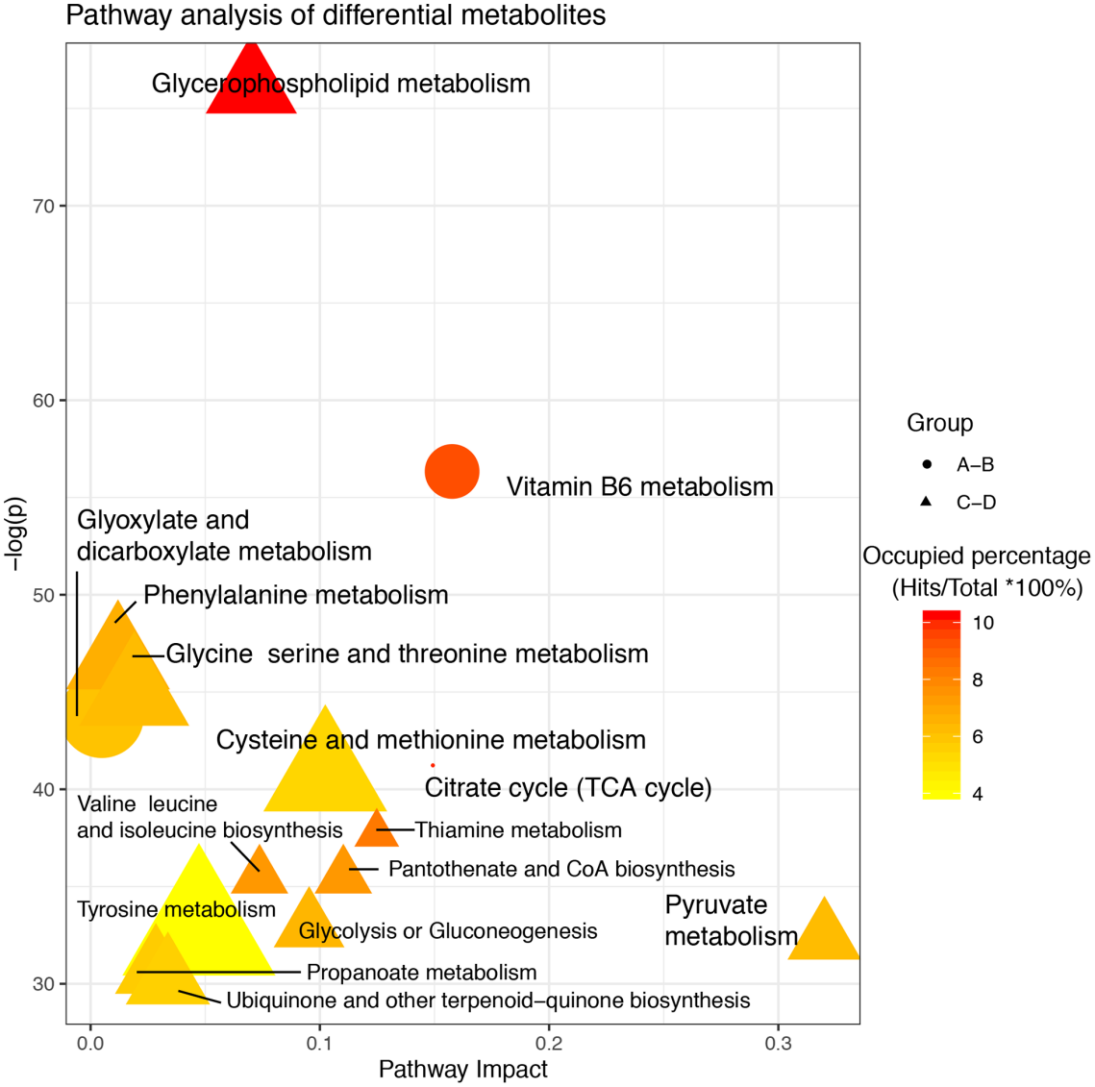
**Pathway analysis of altered metabolites isolated from patients with delayed and enhanced postoperative recovery in the PACU and at 24 h after surgery.** The X-axis represents the pathway impact, and the Y-axis represents the –log (p). Node size indicates metabolites in a specific pathway, and colors indicate the occupied percentage (the hit metabolites occupying a percentage of all metabolites in the whole pathway). Yellow to red represents high to low. The shapes of the nodes represent differential metabolites in different groups. Round represents differential metabolites between A and B; Triangle represents differential metabolites between C and D.

After screening differential metabolites, the enriched pathways were further analyzed (Figure 3). In groups A–B, glyoxylic acid and dicarboxylic acid metabolism, vitamin B6 metabolism, and pyruvate metabolism were identified as significantly enriched pathways. Two of these three pathways showed high occupancy percentages (>90%). Notably, glycerophospholipid metabolism was the most significantly enriched pathway in groups C–D and showed the highest occupancy percentage (>90%). The phosphohydroxypyruvic acid selected only in groups C–D was enriched in glycine, serine, and threonine metabolism.

### SVM analysis of blood samples delayed and enhanced postoperative recovery patients

**Figure 4:**
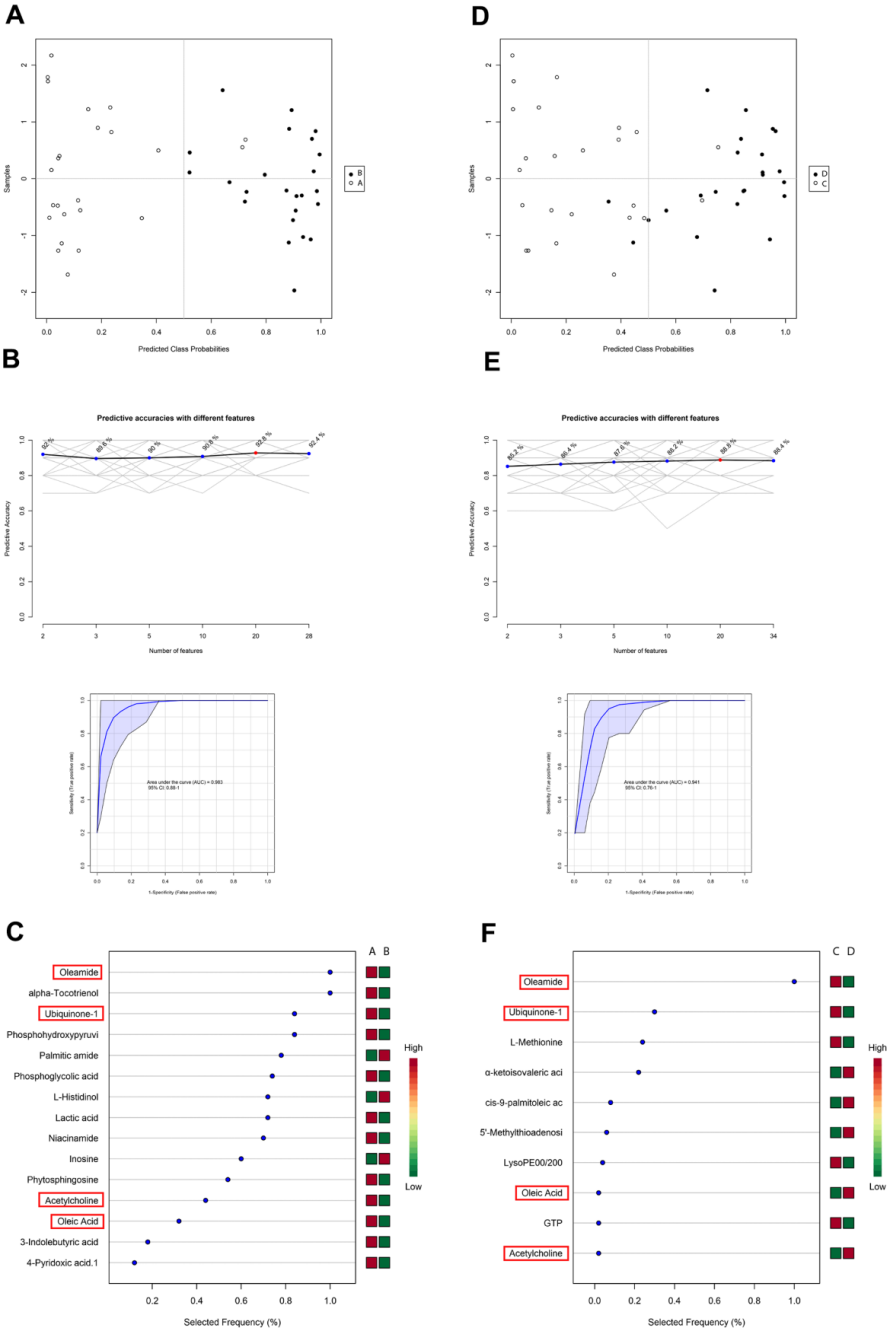
**Support vector machine (SVM) analysis of metabolites in blood samples in the PACU and at 24 h after surgery in patients with delayed and enhanced postoperative recovery.** (**A**) SVM classification of samples in groups (**A**, **B**). (**B**) Specificity, sensitivity, and predictive accuracies of the (**A**, **B**) SVM model. (**C**) Features’ importance ranking of the (**A**, **B**) SVM model. (**D**) SVM classification of samples belonging to groups (**C**, **D**). (**E**) Specificity, sensitivity, and predictive accuracies of the (**C**, **D**) SVM model. (**F**) Features’ importance ranking of the (**C**, **D**) SVM model. The red cubes represent higher importance, and the green cubes represent lower importance in the SVM model.

Identification of differential blood metabolites between the enhanced and delayed postoperative recovery groups suggested that some of the identified metabolites may have applications as biomarkers to predict whether a patient would suffer from poor postoperative recovery after surgery. Therefore, an SVM model was applied to identify the patterns among metabolites. By using these differentiated blood metabolites between group A and B as input features, an SVM model with 22 features was obtained (Figure 4A). An AUC score of 0.983 indicated an effective model, and the prediction accuracy reached 92.8% (Figure 4B). For samples belonging to groups C and D, the optimal SVM model had 10 features (Figure 4D). An AUC score of 0.941 indicated an effective model, and the prediction accuracy reached 88.8% (Figure 4E).

In both the A–B and C–D SVM models, there were four metabolites calculated as important features (Figure 4C and 4F). Oleamide, ubiquinone-1, acetylcholine, and oleic acid could be used to separate patients exhibiting enhanced and delayed postoperative recovery both in the PACU and at 24 hours after surgery. Thus, these metabolites could be used as potential biomarkers to predict patients’ postoperative recovery state according to the SVM results. Among all these features, oleamide had the highest selected frequency, and ubiquinone-1 also ranked among the top 3 in both models.

There were 28 differential metabolites between groups A and B; 22 of these metabolites could be used as important features in the A–B SVM model. Among the nine metabolites showing similar variation trends compared with those in the C–D model, six were identified as SVM features. For the C–B model, 10 of 34 differential metabolites were used as important features in the SVM model, whereas five feature metabolites had similar variation trends compared with groups A–B among the 21 total metabolites.

## DISCUSSION

The recovery rate of GI function is an essential factor reflecting postoperative recovery in patients undergoing colorectal surgery. Morbidity and mortality are both influenced by postoperative care. Although GDFT guided by the ERAS program is helpful for postoperative recovery in most situations and GDFT under SVV guidance is an appropriate strategy for fluid management during and after colorectal surgery, some cases show opposite trends. Therefore, it is critical to identify methods to evaluate and predict potential GI function recovery during the early postoperative period such that countermeasures may be applied to enhance prognosis. In this study, all patients underwent colorectal surgery with GDFT under SVV guidance, and patients were grouped according to different clinical outcomes and compared based on variations in plasma metabolites.

Disease initiation and progression can also lead to changes in metabolic substances or metabolic pathways. To explore complex and dynamic living systems, omics analyses have recently been established [15]. Metabolomics, defined as “the quantitative measurement of the dynamic multiparametric metabolic response of living systems to pathophysiological stimuli or genetic modification” [16], plays an essential role in disease prediction and diagnosis. Anesthesia and surgery are both critical factors that affect postoperative GI function. Moreover, different metabolic responses among patients may influence these outcomes. Therefore, in this study, we explored differences in metabolites between patients with delayed and enhanced postoperative GI function recovery to evaluate potential differences in biological responses. Samples isolated in the PACU reflected the immediate response of metabolism after anesthesia and surgery, whereas sample collected at 24 hours after operation provided insights into more long-term changes and trends in metabolic alterations. We evaluated blood samples from 24 patients in both the delayed and enhanced postoperative recovery groups and found significant differential metabolites at both time points in both groups, allowing classification with the help of an SVM model. Finally, we identified four potential biomarkers that could be used to predict postoperative recovery both in the PACU and at 24 hours after surgery.

Differential metabolites may not only serve as biomarkers for prediction but may also suggest underlying metabolic mechanisms of GI function recovery. In our analysis of metabolic differences, 28 significant differential metabolites were identified in blood samples from patients with delayed and enhanced postoperative recovery in the PACU and 34 significant differential metabolites were identified at 24 hours after surgery. Although nine and 21 metabolites in blood samples from the PACU and at 24 hours after surgery showed similar variation trends, only phosphohydroxypyruvic acid showed significant variations. Phosphohydroxypyruvic acid is an intermediate product generated during the transformation from pyruvate to serine and is therefore related to glycine, serine, and threonine metabolism [17]. Pyruvate affects energy production, which is easily influenced by fluid management, and is reflected in phosphohydroxypyruvic acid levels [18]. Vitamin B6 is helpful for the postoperative recovery of patients with cerebral aneurysm [19], and its importance in patients with colorectal cancer suggests that this compound may be beneficial in general postoperative recovery, i.e., for patients undergoing different types of surgeries. Additionally, the tricarboxylic acid (TCA) cycle is activated in energy supplement-related processes, which can be affected by liquid management [20].

In order to distinguish patients who were responsive to GDFT, we used an SVM model to classify patients according to the time of postoperative recovery. Using differential metabolites from samples collected in the PACU and 24 hours after surgery, we identified four metabolites that could have applications as biomarkers. Although oleamide, ubiquinone-1, and oleic acid were not related to enrichment of differential metabolite pathways, these compounds were associated with glycerophospholipid metabolism, including acetylcholine. Moreover, this pathway was highly significant pathway in groups C–D. Thus, glycerophospholipid metabolism could promote intestinal function recovery, amino acid metabolism, and the TCA cycle [21]. Amino acid metabolism promotes protein production, maintains TCA cycle anaplerosis, enhances antioxidant capacity, and stabilizes lipid membranes by suppressing glycerophospholipid metabolism. These biological processes facilitate postoperative recovery and improve outcomes.

Few studies have evaluated metabolic differences in patients undergoing colorectal surgery with different degrees of postoperative GI function recovery. In this study, we used SVV-guided GDFT as an intra-operative fluid strategy and explored the correlations between early postoperative metabolomic differences and postoperative intestinal function. However, we only included patients who underwent colorectal surgery, not whole abdominal surgery (e.g., cesarean section and others). Importantly, not all abdominal surgeries involve the gastrointestinal tract, and different types of surgeries may result in different procedures for recovery of intestinal function. Colorectal surgery is known to alter the integrity of the intestinal tract, and the recovery of intestinal function may mainly depend on mechanisms mediated by the intestinal tract itself. The PLS-DA model used in this study to identify significant differential metabolites showed low prediction accuracy; thus, some metabolites with the same variation tendencies in groups A–B and C–D were not recognized as significantly different. However, the model showed good performance without excessive lifting, indicating that although we could ignore some significant differential metabolites, the selected metabolites were reliable.

In summary, we proposed a novel method to predict postoperative recovery of GI function using metabolomics. From only 10 metabolites isolated from blood samples collected 24 hours after surgery blood samples or 22 metabolites from blood samples collected in the PACU, we may predict the patient’s possible long-term postoperative recovery condition of GI function. Four potential biomarkers were identified. Further studies are needed to evaluate whether these biomarkers may have potential therapeutic applications in promoting postoperative GI function.

## MATERIALS AND METHODS

### Study design and participants

This was a single-center, prospective, observational cohort study. Two parallel groups (enhanced and delayed recovery) were enrolled according to the postoperative time of flatus. Patients who had undergone elective surgery for colorectal cancer resection were enrolled from December 2018 to October 2019 at Shanghai Changhai Hospital, Second Military Medical University. Written informed consent was obtained from all participants on the day before surgery. Patients who met any of the following criteria were excluded: ASA IV or V; age < 18 years or > 70 years; fasting time < 8 hours or > 20 hours; pre-operative ileus; body mass index (BMI) < 18 kg/m^2^ or > 25.0 kg/m^2^; presence of cardiovascular diseases, such as coronary artery or valvular diseases, New York Heart Association Functional Classification class II or higher heart failure, and arrhythmia; presence of metabolic diseases, such as severe diabetes, ketoacidosis, hyperosmolar syndrome, or long-term hypoglycemia; presence of systemic immune disease; presence of liver and kidney dysfunction; presence of chronic obstructive pulmonary disease; and refusal to participate in the study or presence of mental illness and inability to communicate correctly. In addition, because of the pneumoperitoneum in laparoscopic surgery may affect the change of chest pressure, and the accuracy of SVV monitoring may be influenced, thereby the laparoscopic colorectal surgeries were also excluded.

### Anesthesia

General anesthesia was induced by midazolam (0.04 mg/kg), propofol (1–2.5 mg/kg), fentanyl (2.5–6 μg/kg), cis-atracurium (0.2–0.3 mg/kg), and dexamethasone (10 mg). Anesthesia was maintained by inhalation of sevoflurane (0.8–1.3 MAC) with intermittent supplementation using other auxiliary drugs, such as remifentanyl (0.1–0.2 μg/kg/min), dexmedetomidine (0.2–0.5 μg/kg/h), cis-atracurium, and fentanyl. To accelerate postoperative extubation, inhalation was discontinued, and total intravenous anesthesia by propofol (100–200 μg/kg/min) was applied before abdominal closure. Patients were ventilated in a volume-controlled mode with a tidal volume of 8 mL/kg (air: 40–100% oxygen). The respiratory rate was set to maintain the PaCO_2_ between 35 and 45 mmHg to improve the operating conditions, and the positive end-expiratory pressure was set at 6–8 mL H_2_O when necessary. For intra-operative analgesia, each patient received bilateral transverse abdominal fascia nerve blocks containing 20–30 mL ropivacaine (0.375%) after induction.

### Fluid management and hemodynamic monitoring

On arrival at the operating room, patients received intravenous ringer lactate (5 mL/kg), and hemodynamic parameters were recorded as the baseline. After induction of anesthesia, a 20-G radial arterial line was inserted and connected to an electrocardiogram monitor and fourth-generation Vigileo/Flotrac system (Edwards Lifesciences, Irvine, CA, USA) to determine changes in arterial blood pressure waveforms, SVVs, and cardiac indexes. A double-lumen 5.5-Fr catheter (arrow central venous catheter; Teleflex Life Sciences Ltd., Athlone, Ireland) was inserted into the right internal jugular vein for colloid supplementation. A continuous infusion of ringer lactate was given through peripheral veins during the operation at a rate of 3 mL/kg/h. Colloid (hydroxyethyl starch 130/0.4 sodium chloride injection; Fresenius Kabi, USA) management was conducted under the guidance of the Vigileo/Flotrac system. If the SVV changed by more than 10% 2–3 consecutive times, the colloid was injected via the central vein at 4 mL/kg. After 5 min, SVV changes were observed again. Vasoactive agents were given according to changes in intra-operative hemodynamics based on the recommendations of the anesthesiologist. Transfusions were applied according to recent guidelines [22].

### Data collection

The study was designed as a two-arm, parallel, prospective, observational cohort. The primary endpoint of the study was the timepoint of flatus and differences in blood metabolomics between the two groups. Hemodynamic parameters at different timepoints, surgical resection methods, total intra-operative time, and perioperative fluid volume were recorded. Additionally, the number of hemodynamic fluctuations, such as hypotension or hypertension, was collected. The timepoints of flatus and feeding were recorded, and the time of flatus was used as an indicator of postoperative recovery. The occurrence of flatus 72 hours after surgery was identified as delayed postoperative recovery. The length of hospital stay and duration of postoperative complications were calculated.

### Blood sample collection

Blood samples were collected from all patients at PACU admission and 24 hours after surgery. Blood was collected into heparinized tubes, and plasma was immediately separated by centrifugation at 3000 rpm for 10 min and stored at –80° C until metabolomics analysis.

### Liquid chromatography (LC)/mass spectrometry (MS) analysis

LC/MS analysis was carried on an Ultimate 3000LC Q Exactive platform (Thermo, USA). The parameters for electron spray ionization (ESI)+ and ESI- ion modes were as follows: heater temperature, 300° C; sheath gas flow rate, 45 arb; auxiliary gas flow rate, 15 arb; sweep gas flow rate, 1 arb; spray voltage, 3.0 kV; capillary temperature, 350° C; S-Lens RF level, 30%. The data were analyzed using feature extraction and preprocessed with Compound Discoverer software (Thermo). In total, 2015 features in ESI+ ion mode and 1601 features in ESI- ion mode were evaluated, and editing of data was performed with the Multivariate Analysis (MVA) feature of SIMCA-P software (Umetrics AB, Umea, Sweden).

### Biomarker identification

A supervised learning model was built by a support vector machine (SVM) to classify the delayed and enhanced postoperative recovery groups. Finally, we used radial as SVM kernel function under type C classification and selected 22 features (A–B) and 10 features (C–D) to construct the two SVM models separately. Under these parameters, the predictive accuracies reached 92.8% and 88.8%, and the area under the curve (AUC) values were 0.983 and 0.941 for groups A–B and C–D, respectively, indicating that the model was effective.

### Statistical analysis

Statistical analysis was performed using GraphPad Prism version 8.0 (San Diego, CA, USA). Baseline demographics and postoperative complications were evaluated using Pearson’s chi-square tests for categorical variables, with Fisher’s exact test for small counts. Analysis of variance with Tukey’s correction was used for continuous data. Results with *P* values less than 0.05 were considered statistically significant.

### Ethics approval and consent to participate

The study was approved by the appropriate Institutional Review Board (IRB) and Changhai Hospital Ethics Committee (Shanghai, China; approval no. CHEC2018-080) and was registered at the Chinese Clinical Trial Registry (http://www.chictr.org.cn/index.aspx; identifier: ChiCTR1800015899). Written informed consent was obtained from all patients or legal guardians.

### Consent for publication

Patients gave consent for their personal or clinical details to be published in this study. A copy of the written consent is available for review by the Editor-in-Chief of this journal.

### Availability of data and materials

The datasets used and/or analyzed during the current study are available from the corresponding author on reasonable request.

## Supplementary Material

Supplementary Figure 1

Supplementary Tables
