# Supplementary material for: Blood metabolomic profiling predicts postoperative gastrointestinal function of colorectal surgical patients under the guidance of goal-directed fluid therapy
Source: Aging (Albany NY). 2021 Mar 10;13(6):8929–43. doi: 10.18632/aging.202711 (PMC8034902; doi:10.18632/aging.202711)
Supplement: Supplementary Figure 1 [file aging-13-202711-s001.pdf]

SUPPLEMENTARY FIGURE

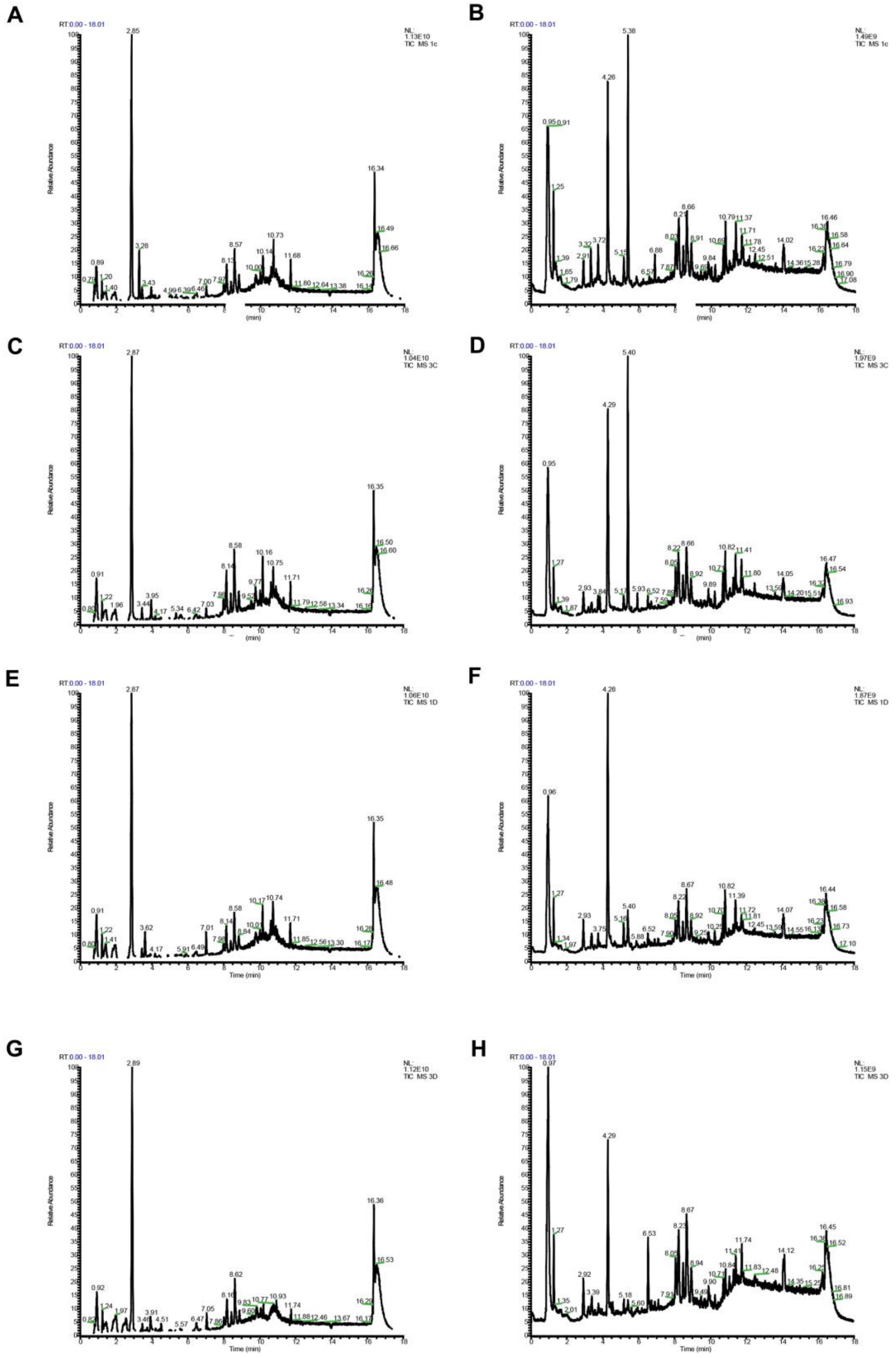

Supplementary Figure 1. (**A**) Typical ion chromatogram from group A (ESI+). (**B**) Typical ion chromatogram from group A (ESI-). (**C**) Typical ion chromatogram from group B (ESI+). (**D**) Typical ion chromatogram from group B (ESI-). (**E**) Typical ion chromatogram from group C (ESI+). (**F**) Typical ion chromatogram from group C (ESI-). (**F**) Typical ion chromatogram from group D (ESI+). (**G**) Typical ion chromatogram from group D (ESI-).
