# Supplementary material for: Blood metabolomic profiling predicts postoperative gastrointestinal function of colorectal surgical patients under the guidance of goal-directed fluid therapy
Source: Aging (Albany NY). 2021 Mar 10;13(6):8929–43. doi: 10.18632/aging.202711 (PMC8034902; doi:10.18632/aging.202711)
Supplement: Supplementary Tables [file aging-13-202711-s002.pdf]

SUPPLEMENTARY TABLES

Supplementary Table 1. Differentiating metabolites between PACU and 24h after surgery groups in delayed postoperative recovery patients.

RT [min]	Molecular Weight	Name	Δppm	VIP	T-test	Fold change(A/B)	ESI
10.126	255.25679	*Palmitic amide	2	1.64014	0.046	-0.612	+
3.165	189.08319	*3-Indolepropionic acid	22	2.29101	0.004	-0.529	+
3.853	203.09885	*3-Indolebutyric acid	21	1.80197	0.027	-0.5111	+
2.762	158.04094	*Allantoin	19	2.27357	0.004	-0.450	+
2.76	175.06743	*3-Indoleacetic Acid	23	2.26836	0.004	-0.449	+
16.355	183.979	*Phosphohydroxypyruvic acid	9	1.79561	0.028	-0.307	+
0.988	165.04636	*Methionine sulfoxide	2	2.33843	0.003	-0.304	+
7.196	250.12146	Ubiquinone-1	3	2.59118	0.001	0.101	+
3.742	165.07982	L-Phenylalanine	4	1.89436	0.020	0.183	+
16.605	155.98377	Phosphoglycolic acid	9	2.27868	0.004	0.204	+
2.554	141.0909	L-Histidinol	4	2.44867	0.002	0.208	+
7.391	317.29412	Phytosphingosine	3	2.47505	0.001	0.226	+
8.195	424.3398	α-Tocotrienol	13	1.88778	0.021	0.328	+
8.983	282.25675	Oleic Acid	3	2.63855	0.001	0.363	+
1.759	122.04866	Niacinamide	5	3.15295	<0.001	0.472	+
2.82	145.11081	Acetylcholine	3	1.63165	0.048	0.543	+
3.827	268.08348	Inosine	10	2.4605	0.002	0.579	+
8.391	281.27254	Oleamide	2	4.26071	<0.001	0.687	+
1.233	167.059	Pyridoxal	4	2.0244	0.013	1.314	+
1.307	183.05395	4-Pyridoxic acid	4	2.02674	0.013	2.563	+
1.269	90.02981	*Lactic acid	21	2.06542	0.028	-0.405	-
8.664	256.23927	*Palmitic acid	3	2.29149	0.014	-0.128	-
1.269	148.03545	Citramalic acid	11	2.05027	0.030	0.209	-
0.976	192.02554	Citric acid	7	1.86695	0.049	0.352	-
0.972	146.01999	Oxoglutaric acid	10	1.94404	0.040	0.401	-
0.97	120.04067	Purine	24	2.10725	0.025	0.638	-
4.934	515.29005	Taurocholic acid	3	2.2569	0.016	0.829	-
1.355	183.05169	4-Pyridoxic acid	7	2.39692	0.010	2.812	-

Note: Fold change (A/B)=log2(mean(A)/mean(B)), - represents decreasing compared of A to B; The same variation trendency in A-B and C-D are marked as with *.

Supplemental Table 2. Differentiating metabolites between PACU and 24h after surgery groups in enhanced postoperative recovery patients.

RT [min]	Molecular Weight	Name	Δppm	VIP	T-test	Fold change(C/D)	ESI
10.589	316.2413	*Pregnenolone	3	2.03901	0.001	-0.783	+
11.031	286.23073	*Retinol	3	2.7516	<0.001	-0.761	+
0.946	149.05166	*L-Methionine	4	1.31269	0.045	-0.582	+
16.633	183.97899	*Phosphohydroxypyruvic acid	9	1.66377	0.010	-0.513	+
7.998	183.06665	*Phosphocholine	3	2.28264	<0.001	-0.479	+
1.759	122.04866	*Niacinamide	5	1.28864	0.049	-0.470	+
9.394	481.31862	*LysoPC(15:0)	3	2.03494	0.001	-0.371	+
8.061	103.10025	*Choline	5	1.49747	0.021	-0.369	+
2.832	169.05346	*Phosphorylcholine	18	1.50309	0.021	-0.368	+
8.405	453.28721	*LysoPE(0:0/16:0)	3	1.83632	0.004	-0.358	+
9.167	509.35014	*LysoPE(0:0/20:0)	3	1.85395	0.004	-0.355	+
7.569	467.30315	*LysoPC(14:0)	4	1.49096	0.022	-0.352	+
2.775	297.09094	*5'-Methylthioadenosine	4	2.14695	<0.001	-0.333	+
8.391	281.27254	*Oleamide	2	3.27455	<0.001	-0.315	+
8.33	254.22522	*cis-9-palmitoleic acid	2	1.50032	0.021	-0.286	+
9.794	523.36576	*LysoPC(18:0)	3	1.36424	0.037	-0.195	+
0.956	181.07476	*L-Tyrosine	5	1.37243	0.036	-0.189	+
1.236	164.04809	*4-Hydroxycinnamic acid	4	1.41536	0.030	-0.162	+
8.602	495.33424	*LysoPC(16:0)	3	1.29037	0.049	-0.157	+
10.064	314.24667	Octadecanedioic acid	3	1.32469	0.043	0.057	+
7.196	250.12146	Ubiquinone-1	3	2.11399	<0.001	0.105	+
9.079	282.2567	Oleic Acid	2	2.57534	<0.001	0.283	+
0.806	165.06537	7-Methylguanine	2	1.96065	0.002	0.284	+
1.206	116.04795	α-ketoisovaleric acid	5	1.379	0.035	0.292	+
2.82	145.11081	Acetylcholine	3	1.6783	0.009	0.309	+
1.213	102.03242	Acetoacetic acid	6	1.87345	0.003	0.336	+
0.809	123.03454	Niacin	20	2.24416	<0.001	0.366	+
8.457	453.28449	*LysoPE(0:0/16:0)	2	1.71348	0.020	-0.289	-
9.455	481.31564	*LysoPC(15:0)	2	2.0867	0.004	-0.376	-
6.469	522.98842	GTP	4	1.87985	0.010	0.256	-
0.982	88.01421	Pyruvate	20	1.48087	0.046	0.662	-
1.269	90.02981	Lactic acid	21	1.53019	0.039	0.634	-
3.275	264.11034	Thiamine	22	1.51916	0.041	0.679	-
0.92	120.04071	Purine	24	1.73127	0.019	0.280	-

Note: Fold change (C/D)=log2(mean(C)/mean(D)), - represents decreasing compared of C to D; The same variation tendency in A-B and C-D are marked with *.
